# MS-Based Proteomics of Body Fluids: The End of the Beginning

**DOI:** 10.1016/j.mcpro.2023.100577

**Published:** 2023-05-19

**Authors:** Jakob M. Bader, Vincent Albrecht, Matthias Mann

**Affiliations:** 1 Department of Proteomics and Signal Transduction, Max Planck Institute of Biochemistry, Martinsried, Germany; 2 Faculty of Health Sciences, Novo Nordisk Foundation Center for Protein Research, University of Copenhagen, Copenhagen, Denmark

**Keywords:** biomarkers, clinical tests, body fluids, rectangular strategy, reference channel

## Abstract

Accurate biomarkers are a crucial and necessary precondition for precision medicine, yet existing ones are often unspecific and new ones have been very slow to enter the clinic. Mass spectrometry (MS)-based proteomics excels by its untargeted nature, specificity of identification, and quantification, making it an ideal technology for biomarker discovery and routine measurement. It has unique attributes compared to affinity binder technologies, such as OLINK Proximity Extension Assay and SOMAscan. In in a previous review in 2017, we described technological and conceptual limitations that had held back success. We proposed a ‘rectangular strategy’ to better separate true biomarkers by minimizing cohort-specific effects. Today, this has converged with advances in MS-based proteomics technology, such as increased sample throughput, depth of identification, and quantification. As a result, biomarker discovery studies have become more successful, producing biomarker candidates that withstand independent verification and, in some cases, already outperform state-of-the-art clinical assays. We summarize developments over the last years, including the benefits of large and independent cohorts, which are necessary for clinical acceptance. Shorter gradients, new scan modes, and multiplexing are about to drastically increase throughput, cross-study integration, and quantification, including proxies for absolute levels. We have found that multiprotein panels are inherently more robust than current single analyte tests and better capture the complexity of human phenotypes. Routine MS measurement in the clinic is fast becoming a viable option. The full set of proteins in a body fluid (global proteome) is the most important reference and the best process control. Additionally, it increasingly has all the information that could be obtained from targeted analysis although the latter may be the most straightforward way to enter regular use. Many challenges remain, not least of a regulatory and ethical nature, but the outlook for MS-based clinical applications has never been brighter.

## Highlights

• Technological developments in MS-based biomarker discovery.
• Successful use cases for MS-based proteomics in body fluids in biomarker discovery.
• High-throughput capabilities now enable the analysis of large and independent cohorts.
• Multi-protein biomarker panels as the future of diagnostics.
• Emerging focus on downstream aspects such as data protection and ethics.

## Graphical Abstract

**Figure:**
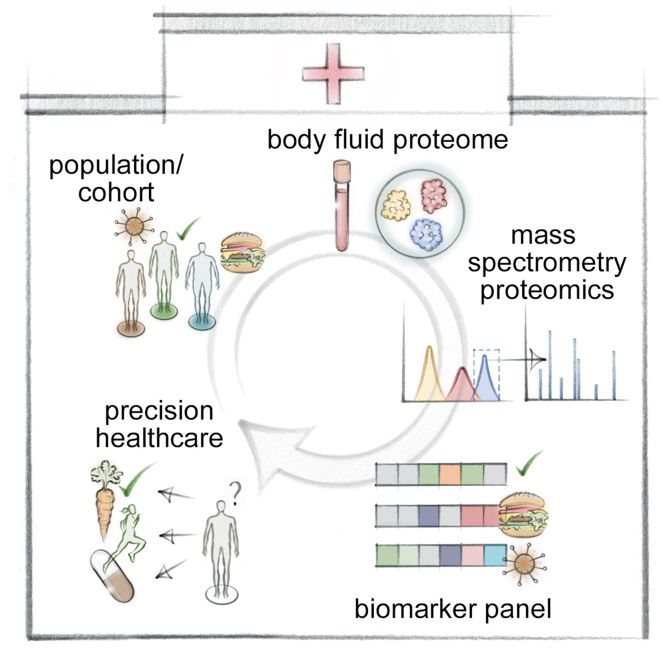

## In Brief

Mass spectrometry is by its nature well suited for identification of body fluid biomarkers and their routine measurement but has been held back by technological and conceptual constraints. Here, we show that improvements in throughput, streamlining, robustness, and study design are progressively overcoming these constraints and have recently enabled promising success stories. Cutting-edge scan modes, multiplexing, and sample preparation promise to further enhance the technology, which we envision will finally bring MS-based proteomics to the clinic.

A biomarker is a “defined characteristic that is measured as an indicator of normal biological processes, pathogenic ones, or a response to an exposure or intervention” (1, 2). It is hard to overstate the importance of biomarkers in medicine and the harm that is done by their absence, leading modern medicine to “fly blind” in all too many cases. Biomarkers can be applied in a multitude of scenarios, guiding health care actions with the aim of optimal patient benefit. Typical cases include the (differential) diagnosis of diseases, screening of populations at risk and assessment of disease complications and risks. Additionally, they ideally help to predict disease trajectories, select appropriate therapies as well as evaluate the efficacy and safety of novel drugs and therapies. In fact, biomarkers reportedly double the probability of success of clinical trials in drug development (3). Body fluids are the prime source of biomarkers given their accessibility and that they reflect processes in organs that adjoin them. The most prominent one is blood (plasma or serum), which—given its immediate contact with almost all organs of the body—is thought to reflect a person’s entire health status. More localized body fluids such as cerebrospinal fluid (CSF), urine, saliva, and nasal mucus report on alterations of adjacent organs such as brain, kidney, and mouth/respiratory tract, respectively.

Proteins are major components of body fluids and are used widely for contemporary diagnostic testing, for instance, they constitute more than 40% of blood analyses (2). Inspired by proteins being vital for all aspects of life, an entire field of body fluid proteomics has emerged with the overarching goal of discovering protein biomarkers and enabling their translation into routine clinical applications (4, 5). This quest has been going on for more than 2 decades and has indeed led to an inflation of “biomarker candidates”((6, 7)). However, these candidates have rarely been independently confirmed or translated into clinical practice. Twenty years ago, upregulation of Beta-2-microglobulin and downregulation of Apolipoprotein A1, Transthyretin/Prealbumin, and Transferrin were reported to better predict ovarian cancer than CA-125/Mucin-16 alone, and this led to an FDA-approved test (OVA1) to be used in conjunction with other diagnostic tools (8, 9, 10). However, to our knowledge, this has remained a “one off.” The limited number of biomarkers that have received regulatory approval is not unique to MS-based proteomics. As of 2013, only around two markers per year have been approved, combining all biomarker types and discovery technologies (7), and this has not yet improved significantly. More than half of the clinical studies for cancer biomarkers are phase one studies while with a mere 0.2% in phase 4 or 5, illustrating the daunting tasks involved in obtaining regulatory approval (https://edrn.nci.nih.gov/data-and-resources/biomarkers/).

In 2017, we reviewed the state of biomarker discovery by plasma proteomics, highlighting pitfalls, and how they might be alleviated (2). One key barrier to success was shortcomings in the overall proteomic pipeline, which in turn impacted non-technological aspects. For instance, low sample throughput entailed small discovery cohorts (typically only dozens of samples) and limited verification in independent cohorts. This resulted in low statistical power in discerning generalizable patterns from cohort-specific effects. Non-robustness and irreproducibility of sample preparation and the analytical setups led to high analytical variability. Furthermore, the low number of proteins detected—especially in blood—had restricted investigation to the most abundant proteins. To alleviate this, many studies employed fractionation and depletion of highly abundant proteins, however, this tended to further reduce the number of samples measured and to increase variability. Apart from this, conceptual issues in study design, sample availability, and issues with sample quality also limited success (2, 11, 12, 13). In the context of biomarkers, the overall metric of success is clinical usefulness with regulatory approval being a major milestone. Three major hurdles to this overall success are the (i) sufficient and “portable,” that is, multicentric, evidence to support large-scale validation, (ii) definition of clinical utility balancing broad applicability and feasibility, and (iii) development of robust assays for clinical application (10).

Fortunately, proteomic technology has progressed tremendously in the past years, overcoming long-standing barriers on the path to that overall goal. Automation using liquid handlers and standardization with quality management systems is starting to enable scaling of sample preparation and improved overall workflow robustness and reproducibility (14, 15). Combined with improved analytical concepts and instrumentation, this has increased throughput of sample measurement leading to larger studies and improved study designs, more accurate results, and more immediate verification. As detailed later in this article, these technological advances have already yielded important biomarker discovery studies in multiple body fluids including plasma, CSF, and urine (15, 16, 17, 18, 19). Given suitable study cohorts, immediate and independent verification can now generate portable evidence to support subsequent large-scale validation. Such validation is still costly and the risk of failure of low-confidence candidates has been a key deterrent. The importance of this aspect is hard to overstate and the lack of verification has also been named as a pivotal barrier elsewhere (7). Regarding clinical utility, proteomic biomarkers attained *via* modern strategies have already provided proof of concept that such utility can be achieved given the right study design, for example, by outperforming state-of-the-art clinical assays (16). Finally, the recent developments now also address barriers of MS toward routine clinical measurement of biomarkers and thus contribute to solving the clinical assay challenge.

Below, we discuss these technological and conceptual developments and their implementation in the past 6 years, focusing on lessons learned, effective practices, and remaining challenges. Many of the examples are from our own group, as we are particularly familiar with them. We also highlight nascent technological advances that are about to enter the biomarker pipeline. Finally, we provide an outlook and recommendations on how these advances might lead to routine acceptance in clinical settings.

## Sample Preparation

### Scalability, Robustness, and Reproducibility

**Fig. 1:**
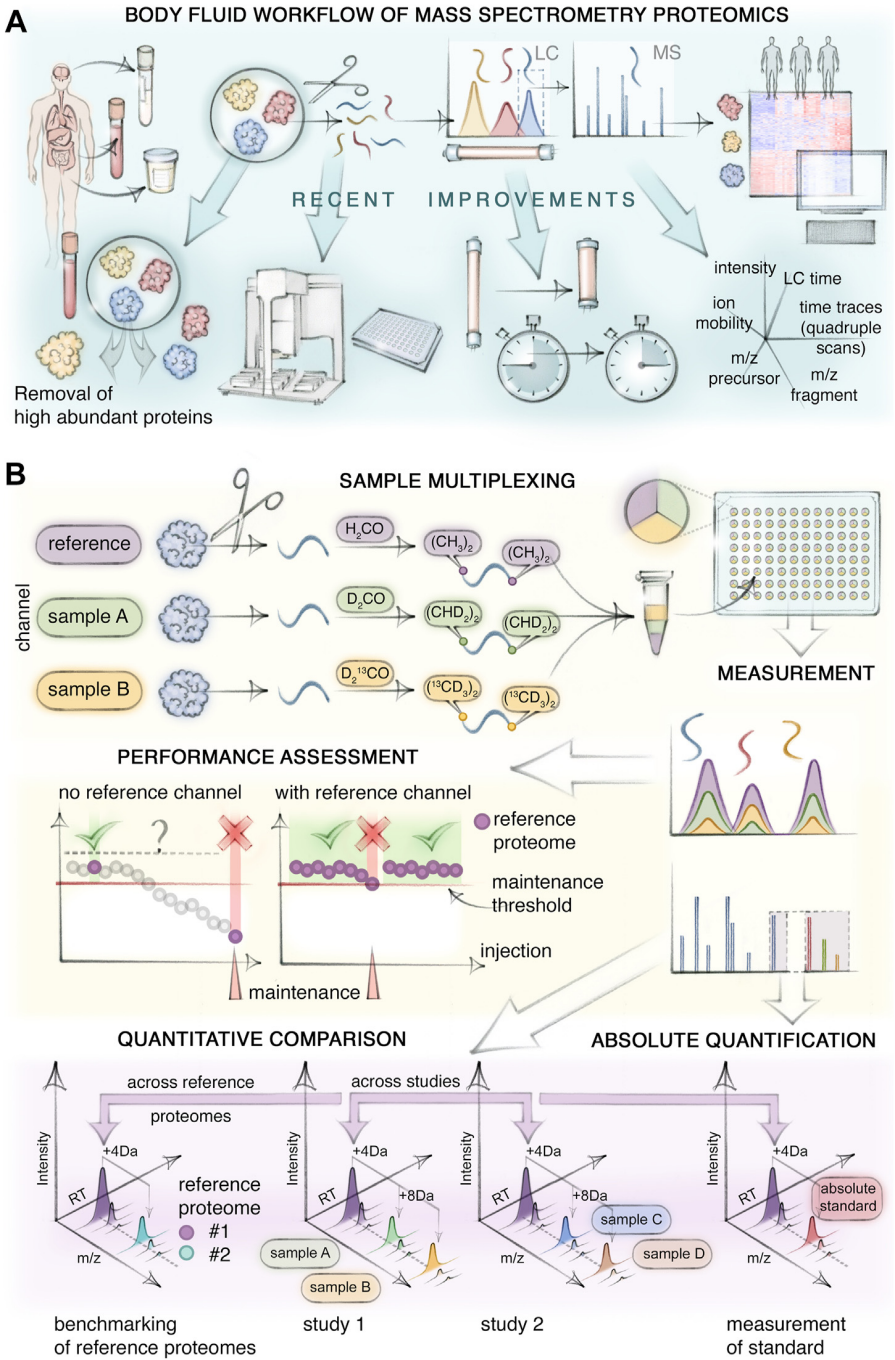
**Recent workflow improvements and the reference channel for body fluid proteomics.***A*, Recent workflow improvements. Removal of highly abundant proteins, *e.g.* albumin in plasma, alleviates the high dynamic range challenge of body fluids. Automation enables robust and high-throughput preparation of samples to obtain clean peptides from proteins. For liquid chromatography separation of peptides, short and high inner diameter columns synergize with robust and fast measurements. MS measurement is aided by additional information such as ion mobility or rapidly moving quadrupole windows. *B*, Multiplexed reference channel. Non-isobaric labeling—here by dimethyl reagents—creates channels recognizable by MS1, permitting the pooling of samples to be measured and the addition of a reference proteome. Channels are related to each other by their characteristic mass shifts. The identifications in the reference proteome in each MS measurement carry over to the sample channels, removing the need for their identification. Additionally, the reference channel enables real-time monitoring of analytical performance. Being identical in all samples, it serves as a bridge for quantitative comparisons across samples of a study or even between studies and laboratories. Different reference proteomes can also be “translated” to each other or ‘harmonized’ by measuring them together. For absolute quantification, a subset of proteins in a reference proteome can be related to absolute standards.

In body fluid proteomics, ensuring consistent, contamination-free, and robust sample preparation quality in manageable time frames is particularly important, given ever-growing study cohorts (Fig. 1*A*). In fact, body fluid applications have driven the quest for these qualities in MS-based proteomics in general, highlighted by an ISO standardized plasma proteomics workflow (15). Today, liquid-handling robots for full or semi-automation have found widespread use. Concomitantly, sample preparation processes have been simplified and streamlined. For instance, while these typically used to involve both a protein digestion and a separate clean-up step prior to MS analysis, the clean up can now be integrated with the digestion step by on-bead precipitation approaches or integrated into the automated online chromatography (20, 21).

### Highly Abundant Protein Challenge

In plasma or serum and to a lesser degree in other body fluids, a handful of highly abundant proteins account for the vast majority of the intensity of quantified peptides, greatly impairing the detection of less abundant ones (18, 19, 22, 23). One long-standing mitigating strategy is the removal (“depletion”) of highly abundant proteins, typically using affinity binders (24). This approach, however, tends to co-deplete other proteins (25, 26), contribute to preanalytical variability, and only provided moderate gains with about 25% more proteins identified in our hands. Furthermore, immunoglobulins, which are typically depleted, can themselves be biomarkers in certain conditions, and depletion of variable levels of these proteins might also introduce difficulty to control co-depletion artifacts. Other common body fluids such as CSF and urine are typically not depleted, but if they are, similar concerns apply, especially given variable blood–brain impairment accompanying some neurodegenerative or neuroinflammatory diseases. That said, for plasma or serum, there are automated solutions for depletion such as those from Agilent, which can be used up to 200 times and aim to make this process reproducible (27).

In a related development, nanoparticles with specific surface chemistries have been used to selectively enrich parts of the proteome. Conceptually building on previous approaches such as ProteoMiner (hexa peptide libraries on beads) (28), the Seer platform has reached a depth of thousands of plasma proteins when adding the results of several fractions (29, 30, 31). Apart from economic considerations, successful adoption will have to await broad preclinical validation. Interestingly, protein precipitation using organic solvents (32) or acids (33, 34, 35, 36, 37) has developed as an alternative enrichment strategy. Recently, several advantages were demonstrated including throughput, cost and time efficiency, minimal batch effects, and reliable detection of plasma proteins at low concentrations (37).

## Liquid Chromatography

Until recently, the liquid chromatography (LC) separation of peptides before the MS analysis was the most variable part of the entire pipeline. High-performance but difficult-to-produce “in house” columns were typically used in proteomics laboratories. There is a clear trend toward commercial ones, as they are more robust and much less variable between laboratories. Micropillar-structured columns (μPAC, Thermo Fisher) may become an attractive alternative to packed columns because of their high resolution and the absence of bead material that may change over time (https://www.thermofisher.com/document-connect/document-connect.html?url=https://assets.thermofisher.com/TFS-Assets%2FCMD%2FTechnical-Notes%2Ftn-000635-ccs-upac-column-robustness-proteomics-tn0006358-en.pdf) (38).

In our own group, until a few years ago, we used long columns (40–50 cm) and gradients (45–100 min) with extensive overhead times (up to 20 min) corresponding to 12 to 24 samples per day (13, 18, 39). Today, we use both shorter columns (8–15 cm) and gradients (21 min, less than 1 min overhead) tremendously increasing throughput (16, 17). While our own columns used to last for tens to hundreds of sample injections, commercial columns in our new setup now last ten times longer and have less performance variability and degradation over time. This is integrated with an HPLC system that pre-mixes the gradients at low pressure and uses a single high-pressure pump to run the gradient at 1 μl/min on the analytical column, concomitantly reducing overhead times (21).

Short gradients with higher flow rates than the typical nanoliter/min ones in proteomics were pioneered on the Sciex instruments (https://sciex.com/content/dam/SCIEX/tech-notes/life-science-research/proteomics/ruo-mkt-02-3637-a/Microflow-SWATH_6600_RUO-MKT-02-3637-A.pdf) but are now well-established across a variety of platforms (40, 41). This approach trades sensitivity for increased robustness and throughput. At a flow rate of a few μl/min, columns have an inner diameter of up to 1 mm, are durable, and yield a good separation in short gradients but require much more input material. At the extreme, very short gradients on classical HPLC columns and flow rates of mL/min enable entire runs in minutes, albeit at the expense of proteome depth (15, 42). Given recent improvements in sensitivity, these approaches are becoming more viable, especially for plasma studies where total protein amount is less of a constraint than in other areas of proteomics.

## Technological Developments in MS

### Wide Adoption of DIA

In recent years, advances in mass spectrometers and acquisition schemes have co-evolved, addressing long-standing challenges. The maturation and widespread adoption of data-independent acquisition (DIA) MS in recent years has marked a significant milestone. This progress has been primarily driven by the development of novel algorithms and high-performance mass spectrometers (43, 44). DIA's inherent ability to sample all precursors at regular intervals results in a more uniform sampling of peptide fragment ions, leading to greater data completeness, enhanced proteome depth, and improved sensitivity (45). As a result, DIA has largely replaced data-dependent acquisition (DDA) methods, except in a few specific workflows like TMT labeling. DIA classically required spectral libraries to match into the acquisition runs. These libraries can be generated experimentally, which often involves sample pooling, protein depletion, and offline fractionation for separate measurements (45, 46). Existing libraries can be used if experimental workflows and analytical setups are sufficiently similar (47). Alternatively, computational approaches are increasingly diminishing the need for experimental libraries. For instance, libraries can be predicted (48, 49) and widely used DIA processing tools permit “library-free” or “direct” DIA data processing, in which a library is derived on the fly from the primary DIA data (50, 51). A number of variants of the basic DIA scheme have been developed (51). For instance, the fast and continuous quadrupole movement of the DIA acquisition window establishes a direct connection between precursors and their fragments, in turn enabling faster analysis speeds and shorter gradients (42). DIA has also been integrated with the ion mobility dimension of separation, in the form of trapped ion mobility spectrometry (diaPASEF), which has become a routine form of measurement for all body fluids in our laboratories (52, 53). Very recently, we introduced the synchro-PASEF scan mode, which extends the conceptual benefits of scanning SWATH to two separation dimensions while also improving quantification due to “precursor slicing” (54). Related scan modes that take advantage of the ion mobility dimension are currently being developed (55, 56).

### Future Developments

For these and other acquisition methods, algorithmic methods urgently need to be developed or improved to take full advantage of them. We expect such software developments to catch up with these new scan modes soon. Once this has happened, these scan modes can be used universally, directly benefitting the depth and quantitative accuracy of body fluid proteomics. MS instrumentation is likewise constantly being improved, for instance with the development of the “Zeno trap,” leading to much-improved fragment detection and quantification and alternative fragmentation modes becoming more readily available (https://sciex.com/products/mass-spectrometers/qtof-systems/zenotof-7600-system.html). Importantly, the direct correlation of precursors and fragments mentioned above will open up for the discovery of modified and otherwise unexpected peptides, including their use as potential biomarkers. Overall, we expect significant improvements in MS technologies and accompanying algorithms in the near future, creating a very deep and solid basis for urinary and CSF proteomes, and going a long way towards deep investigation of the plasma proteome.

## Multiplexing and Reference Channel

### Challenges for Isobaric Labeling of Body Fluids

Sample multiplexing by isotopic labeling is an established approach to increase throughput in proteomics measurements. Tandem Mass Tag (TMT) is currently the most popular variant, wherein each condition is assigned a distinct isobaric label. These labels produce the same mass shifts of precursors but their fragmentation yields channel-specific reporter ions at the same mass for all precursors (57, 58, 59). Today, this allows up to 18 different conditions to be compared in one DDA analysis (60). Apart from foregoing the advantages of DIA, the integration of more samples than are contained in the isobaric set has proven challenging, limiting use in large studies. This is due to the accumulation of missing values across labeling batches of samples due to stochastic sampling in DDA. Furthermore, the principal advantages of multiplexing are in practice offset by the fact that TMT samples are typically extensively fractionated to achieve high proteome coverage (61, 62, 63). Given inherent challenges in the quantification of TMT due to the cross-talk of different peptides in reporter channels and elaborate derivatization requirements, this quantification strategy will be difficult to establish in the clinical routine (64). For cohort measurements, it is therefore currently recommended to use label-free DIA for studies larger than 200 samples (65).

### Non-Isobaric Labeling

Non-isobaric multiplexed DIA (mDIA or plexDIA) (66, 67, 68, 69) is a recent development that promises to combine the advantages of DIA with those of multiplexing. In DDA, non-isobaric multiplexing has been used for many years because of its excellent quantification properties—for instance, in the SILAC method (70). However, the additional complexity of the multiplexed proteome meant that fewer precursors were picked for sequencing, limiting proteomic depth. Multiplexed DIA sidesteps this limitation because all precursors are always fragmented. Indeed, we find proteome depth in triple-labeled mDIA to be nearly the same as in unlabeled samples (69). Thus, this technology now promises high quantification accuracy on the MS1 and MS2 levels while effectively tripling the number of proteins quantified per unit time. A key advantage compared to TMT workflows, especially those with a “carrier” or “booster” channel, is that there are no common low mass reporters that suffer from ratio compression due to co-isolation of other precursors (64, 69, 71). Currently described for three or five channels, multiplexing could be further extended, especially for relatively sparse proteomes such as those of single cells or plasma. In general, however, it may not be useful to extend multiplexing much more because of the very complex MS2 spectra and because additional channels dilute the signal of the individual channel for a given loading capacity. In any case, these exciting advances await dedicated algorithmic developments to take full advantage of them.

### Reference Channel in DIA of Body Fluids

We have used dimethyl labeling because of the ease and completeness of the reaction and because it is very economical (72, 73) (Fig. 1*B*). Importantly, mDIA opens up for the reference channel concept, which could be particularly beneficial for body fluid proteomics. This reference channel consists of a standardized reference sample that is admixed to all samples in a cohort (69). It drastically simplifies and improves identification and quantification, because it is known in great detail and all MS-relevant aspects. Shared peptides from the actual samples are merely versions of the refence peptides that are offset in mass but behave identically in almost all other respects. We have already observed a substantial gain in proteome depth using the reference channel for single-cell proteomics (69, 74). Low-abundant peptides with noisy signals greatly benefit from more abundant peptide signals in the reference channel, resulting in more reliable quantification. In the context of body fluids, we envision that the reference channel would be enhanced by depletion or enrichment to address the issue of highly abundant proteins without increasing analytical variability.

The reference channel can also be used as a built-in quality control of the analytical setup throughout the measurements, thereby replacing injections of standard samples interspersed between the actual samples. If desired, the reference channel can be absolutely quantified for particular biomarkers of interest. This requires a (separate) combined measurement of the reference together with one or more labeled standards, which can even be done retrospectively (Fig. 1*B*). This standard does not need to be confined to one study or one laboratory but could be shared within the entire community. Although very attractive conceptually, much thought will have to be invested in the design of such standards, to which degree they should be universal or disease-specific and how to share them in the community.

## MS-Based Proteomics *Versus* Affinity Binders

Over the last years, affinity binder technologies have been used extensively to measure large clinical plasma or serum cohorts—mainly by Olink’s antibody-based proximity extension assay (PEA) or SomaLogic’s DNA aptamer-based SOMAscan assay, reviewed elsewhere (5, 75, 76). In our opinion, these technologies measure fundamentally different properties and also have different strengths and weaknesses, as outlined below. As of May 2023, PubMed listed about 13,000 publications for the prompt biomarker AND proteomics AND mass spectrometry. By contrast, Olink and SomaLogic report about 1100 and 600 total publications, respectively, on their web pages (https://olink.com/resources-support/publications/, https://somalogic.com/publications/). Many of the MS studies were small and specialized, whereas the affinity binder studies are quite costly but have nevertheless been employed on large cohorts.

### Proteome Depth and Study Size

Affinity binder technologies have had a continuous lead in both proteome depth and sample throughput compared to MS-based proteomics. Earlier versions consisted of disease panels of less than a 100 binders each, which together spanned about a thousand proteins; SomaLogic’s assays covered 1300 proteins in 2017 (77). Today, SomaLogic advertises the detection of 7000 proteins and Olink of 3072 proteins per blood sample (https://somalogic.com/somascan-platform/, https://olink.com/products-services/explore/). Similarly, affinity binder proteomics studies were limited to hundreds of samples a couple of years ago, whereas—given sufficient funds—even population studies of tens of thousands of individuals are now feasible (78, 79). This contrasts to several thousands of samples for MS-based proteomics at most, establishing a standard that MS-based proteomics must now strive to achieve

### Specificity of Detection and Quantification

There are analytical challenges and potential pitfalls with regard to identification and quantification by any binder technology, in particular in multiplexed formats. This is the underlying reason that there are very few multiplexed ELISA assays. Proximity extension assays partly address this issue because the read-out depends on two different antibodies binding the target protein in close proximity, thereby imparting specificity. In contrast, aptamers rely on the exclusive engagement of a unique binder for every target protein. By its nature, MS-based proteomics excels at the specificity of detection and quantification, but its limited depth has made it difficult to evaluate binder technologies by this “gold standard” (80). Absent this, a number of studies have compared SOMAscan to Olink assays, coming to the overall conclusion that there is high quantitative concordance in about a third of shared targeted proteins, moderate correlation in another third, and absence of correlation in the remainder (80, 81, 82). It is not clear that these discrepancies are the fault of one or the other approach and instead, they may reflect the underlying challenges in multiplexed binder assays mentioned earlier. We also note that the MS proteomics community has a long tradition in publicly characterizing all steps of the analytical pipeline including wrong identifications and has developed specific approaches to quantify and manage reliability in body fluid proteomics, such as quality markers to detect platelets or erythrocyte contamination, for example (see below). This aspect has been less prominent in publications using binder approaches and in general, validation data even for individual binders is scarce in the public domain. To the extent this has happened—for instance, by cross-referencing with ELISA assays—results have been inconsistent (80, 81, 82, 83, 84). Moreover, the potential and risks of applying binder assays developed for plasma/serum proteomics to other matrices such as CSF or urine require more investigations.

### Clinically Accepted Biomarker Panels

One would expect that reliably quantifying thousands of plasma proteins in thousands of samples should have resulted in new biomarkers or biomarker panels that would be on course to enter the clinical application. However, it does not appear that affinity binder technologies have solved this unmet need at this point. To our knowledge, potential biomarkers discovered by affinity binder technologies—like MS-based ones—have not reached regulatory approval for routine clinical approval. This may be connected to the above-mentioned challenges in the specificity of detection or it may simply reflect the relative novelty of these technologies, which have not allowed sufficient follow-up of their findings, much less regulatory approvals.

### Perspectives

**Fig. 2:**
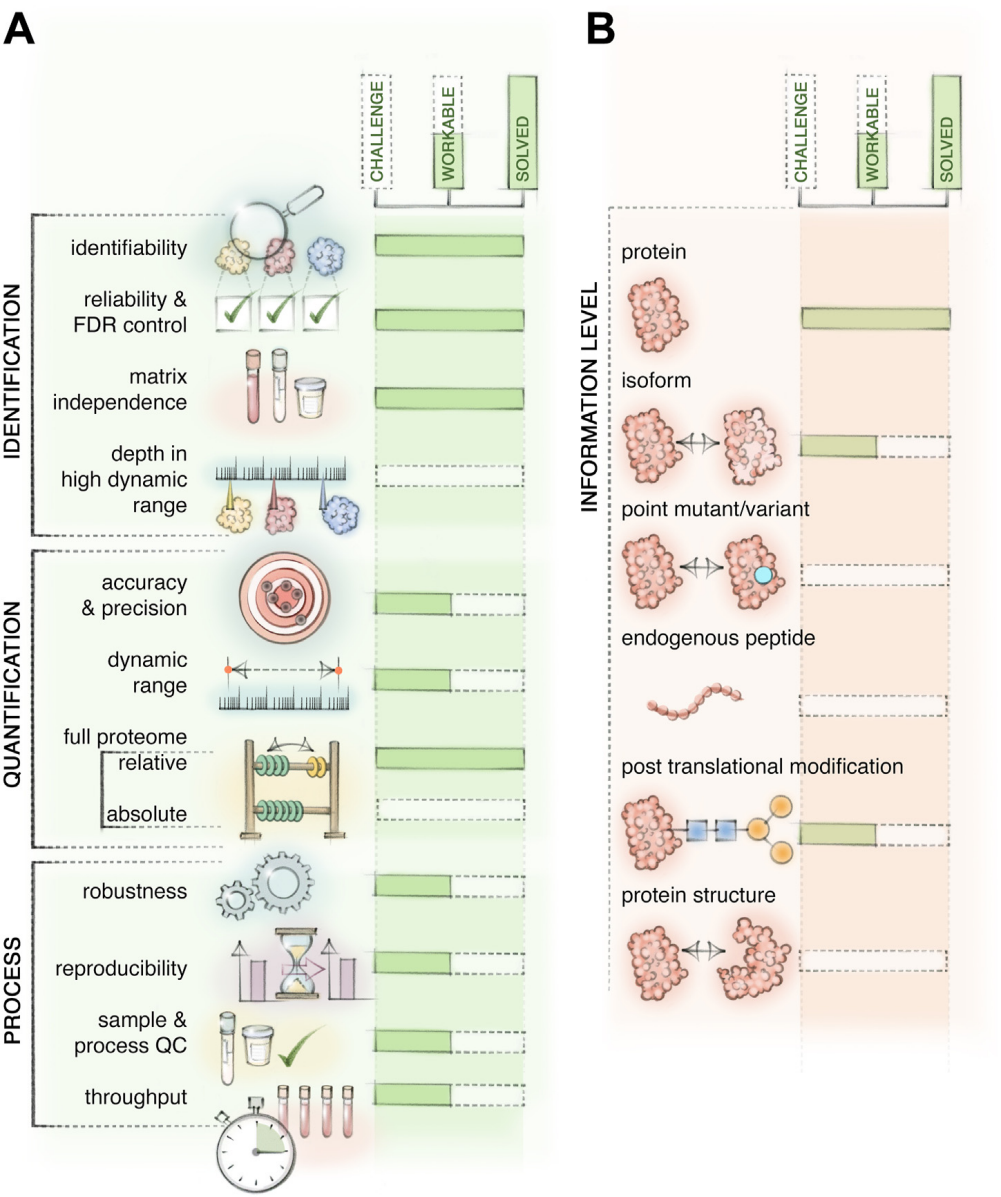
**The state of MS-based body fluid proteomics.***A*, Workflow capabilities of MS proteomics. Identification: MS can conceptionally identify any protein using specific mass patterns, a process that is reliable and can be controlled in terms of false discovery rates (FDR) independent of a body fluid matrix. In practice, the high range of protein abundances in body fluids is a challenge, limiting identifications of very low abundant proteins. Quantification: Accuracy, precisions, and dynamic range of quantitative measurement are the core strength of MS-based proteomics. In practice, the same caveats apply to low abundance proteins, an area that is being addressed by current technological developments. Relative comparisons within and across samples are possible for the entire proteome, while the determination of absolute levels is limited to a few proteins. Process: It is now feasible to measure thousands of samples but the measurement should be tightly controlled for stable calibration, need for cleaning or column replacement. Proteomes inherently contain information to assess samples and processing quality, which can be monitored continuously. Comparing results between laboratories should result in overlapping biomarker candidates, however, a reference proteome (see Fig. 1) would greatly improve this. *B*, Body fluid proteome information produced by MS-based proteomics. Proteins can be precisely identified and quantified, whereas the resolution of splicing isoforms and single amino acid isoforms is also possible in principle but not widely established yet. Likewise, investigation of endogenous peptides is technically feasible but rarely performed on a large scale in body fluids, which also applies to post-translational modifications. Protein structure can be studied on a proteome scale but this is even further off into the future and only realistic for the most abundant proteins in body fluids.

Affinity binder proteomics promise ever greater scaling and may also address current concerns about specificity in the coming years. Conversely, MS is on course to follow suit in proteome depth, sample throughput as well as further improvement in quantification and process aspects (Fig. 2*A*). Moreover, it is suited to study additional proteome aspects less accessible to binder technologies. These include protein isoforms, the peptidome, and the post-translational modification landscape (Fig. 2*B*). As a specific example, glycosylation of secreted proteins is an abundant modification of plasma proteins, and glycation of blood hemoglobin is the gold standard for monitoring blood sugar levels in diabetes, which is readily detected by MS-based proteomics but not currently by the binder assays (85, 86, 87, 88).

## Lessons Learned in Recent Biomarker Studies

### Integration of Discovery and Verification

A successful biomarker development trajectory comprises the phases of discovery, verification in one or more independent cohorts, and validation as a laboratory test leading up to the clinical routine application (4). For more than a decade, the most common proteomic approach was to quantify a large number of proteins using elaborate setups in a small discovery cohort of a low number of subjects. This was supposed to lead to a few biomarker candidates that would be validated on a large scale, perhaps using ELISA assays. Thus, the number of proteins measured decreased from start to finish, whereas the number of participants increased. This approach can be represented as a “triangular strategy,” as opposed to the “rectangular strategy” that we favor (2) and which is also supported by others (5). This is because the triangular strategy has low statistical power due to few initial samples and is vulnerable to sample group biases such as pre-analytical and biological variation, which could result in wrong candidates being carried forward towards validation.

To address these issues, the rectangular strategy aims to measure as many samples in as much depth as possible from at least two independent cohorts in a streamlined format. This results in much greater statistical power and tends to dilute out spurious and study-specific biomarker candidates. It requires a sufficient cohort size of ideally hundreds or thousands of individuals and at minimum a proteome depth that covers the potential biomarkers. Moving toward higher sample numbers in disease contexts further allows the identification of patient subgroups. It has also become clear that longitudinal studies are particularly desirable for proteomics because each individual effectively provides their own control, removing much of the confounder of population heterogeneity.

The rectangular strategy has now enabled biomarker discoveries in multiple body fluids (15, 16, 17, 18, 19). One of these particularly highlighted an advantage or even the necessity of the rectangular strategy: To identify Alzheimer’s disease (AD) biomarkers, we measured three cohorts of about 30 cases and controls each to a depth of 1200 quantified proteins in a unified, single-run setup. This yielded more than 800 statistically significant AD-associated proteins but mostly only in one of the three cohorts. Requiring consistency across all three cohorts reduced this number to a biologically meaningful and manageable set of only 40 proteins. Unbiased analysis by machine learning ranked the tau proteins as the top candidate providing positive control and also uncovered an unexpected glycolytic signature that was independently verified by others (18, 62, 89). Interestingly, the verification rate of the biomarker candidates stabilized when integrating additional cohorts, including replication in independent publications of 70% of the 40 proteins that were consistent across our three cohorts (18, 62). Although successful in guiding biomarker candidate selection to verifiable ones, this experience also draws attention to the shortcomings of the underlying, small cohorts (see below).

### Power of Biomarker Panels

Although attractive in principle, single analyte biomarkers that specifically indicate disease appear to be rare and require large effect sizes that are also resilient to confounders. The combination of multiple biomarkers in a panel is known as “*In Vitro* Diagnostic Multivariate Index Assay,” such as the above-mentioned OVA1 test (90). In our experience, unbiased proteomics usually results in panels of biomarkers that individually have small fold-changes but in combination capture sufficient information in body fluid proteomes to resolve confounders and accurately diagnose disease states. This empirical finding is supported by the notion that the body fluid proteome is multi-dimensional and influenced by factors such as genetics (91, 92, 93, 94), gender (91), age (95), lifestyle (92, 96), prior disease or treatment (97) and that biomarker panels are inherently better suited for to resolve this complexity (2). Our recent experience furthermore suggests that these panels typically comprised 10 to 20 proteins that together allowed patient classification (16, 17, 18, 19).

Diagnosis and prognosis of liver disease is an important clinical need worldwide and one that we contributed to by identifying promising biomarker panels (16). Streamlined analysis of 659 plasma proteomes of a cross-sectional study and an independent cohort again resulted in a relatively small protein signature, which included known and novel potential biomarkers. Measurement of paired liver biopsies furthermore sheds light on the source and trajectories of the biomarkers in the plasma and the tissue. Notably, the MS-derived panels performed equally to or outperformed all clinical state-of-the-art assays for early-stage fibrosis, inflammation, and steatosis. Given the steep improvement curve of the underlying technology of MS-based proteomics, this bodes well for widespread, perhaps even population-wide application.

This liver study also highlighted a general advantage of protein panels *versus* single protein analytes. Our biomarker signature contained the protein prothrombin as a prominent contributor to liver fibrosis diagnostics. ‘Prothrombin time’ is a blood test routinely used in clinical practice to evaluate the coagulation status of patients. However, abnormal levels of this protein do not only reflect liver disease, such as cirrhosis, but also other disorders such as fat malabsorption disseminated intravascular coagulation, and vitamin K deficiency. Clearly, such a marker cannot serve diagnosis by itself and would instead be helped by other proteins that are differentially associated with the disease of interest.

Separately, biomarker associations can also be confounded by sample biases, such as lysis of platelets or erythrocytes in the blood (13). MS-based proteomics readily allows the evaluation of such quality markers in panels, permitting quality control in routine applications.

## The Case for Large, Better Characterized, and Independent Cohorts

### Study Design and Bias

Fundamentally, a study result is valid if it is a reflection of the underlying truth and not obscured by bias. Contrary to the experimental design common to studies of therapeutic intervention, biomarker studies typically have an observational design which inherently makes them more susceptible to bias, for example, due to differences in subject selection (98). Matching of cases and controls is important to reduce such biases but is challenging due to a multitude of confounders such as age, sex, genetic background, lifestyle, metabolic, physical and mental states, treatment, and environmental factors. In practice, it is challenging to obtain such information and additional samples for appropriate matching, forcing researchers to focus on the confounders that are deemed most relevant. However, inappropriate matching has contributed to serious misinterpretation in biomarker associations (99, 100). A common approach to mitigate biases in biomarker studies is a nested case–control design, wherein samples are collected prospectively under equivalent conditions prior to case and control assignment (101, 102). However, it takes many years to collect and perform large studies and in practice, retrospective approaches are still predominant. Nevertheless, a key actionable approach is the study of multicentric cohorts to exclude center-specific biases (103). This creates the “portable evidence” mentioned above (10) that is key to proceeding to validation in large-scale studies. As described earlier, the integration of discovery and verification by the “rectangular strategy” is a promising means to achieve this in proteomic practice. Likewise, an actionable approach to limiting experimental bias is the use of block randomization (104).

### Samples: Suitability, Availability, and Quality

Biomarker studies often suffer from “spectrum bias” which describes a discrepancy between studied subjects and intended generalization (7, 101, 105). For instance, a bias toward severe or advanced disease cases leads to unreliable results in cases typically encountered in the clinic (99, 106). The focus on more extreme cases was in part driven by statistical considerations when cohort size was limiting. However, the increased throughput now addresses this. Another issue goes by the name of “samples of convenience” (7, 107, 108). This refers to the fact that the acquisition of suitable samples for a given clinical question can be difficult, leading to the choice of suboptimal but obtainable samples. Again, biases associated with convenience samples can yield false biomarker associations (109). A 2013 analysis of biomarker study failures found that obtaining samples is typically unsystematic and in practice often determined by established contacts with sample-providing collaborators (7). This could be alleviated by a centralized infrastructure to turn to for appropriate samples or for information on which institutions to approach, but this has not yet happened. Moreover, variations in sample quality and associated biases should be assessed to prevent spurious biomarker associations (13). However, samples that are well-characterized, free of spectrum and quality bias may be considered too precious, in particular for exploratory discovery studies (101). Fortunately, MS-based proteomics requires very little sample material, for example, only a few microliters of blood plasma or CSF (14, 18).

### Clinical Data, Ground Truth, and Subject Selection Criteria

As the proteomics pipeline has matured, it has become clear that not only the samples but also the associated clinical data are crucial factors for determining the success of any proteomic biomarker investigation. For instance, the apparent performance of a novel protein marker panel will always be limited by the quality of the ‘ground truth’. Even a perfect biomarker will produce an apparently wrong prediction if some patients were misdiagnosed, to begin with. In our experience, this is not just a theoretical but a real concern. For instance, in liver disease, pathology assessment of liver biopsies is considered the ground truth but this procedure also has an error rate that puts an upper limit on our success rate (16). In our AD study, there was no single ground truth because neuropathological brain assessment, the overall gold standard for AD diagnosis, was not available for these patients. Instead, clinical AD diagnoses were initially made according to local but inconsistent criteria of the cohort centers (18). Applying uniform AD assignment based on currently accepted CSF biomarker cutoffs improved cross-cohort concordance of proteomic signatures of AD in that case. One cohort still stood out by having a weak separation of AD and controls. Interestingly, an 11-protein panel suggested re-classification of some subjects for which there later turned out to be independent support from the re-examination of patient journals. However, without the underlying ground truth, we could not prove that our AD re-assignment was correct and should have made our success rate even higher. Overall, in our experience, it is easier to standardize by biochemical selection criteria than symptomatic ones when given the chance.

### Large Cohorts Synergize With the Rectangular Strategy

**Fig. 3:**
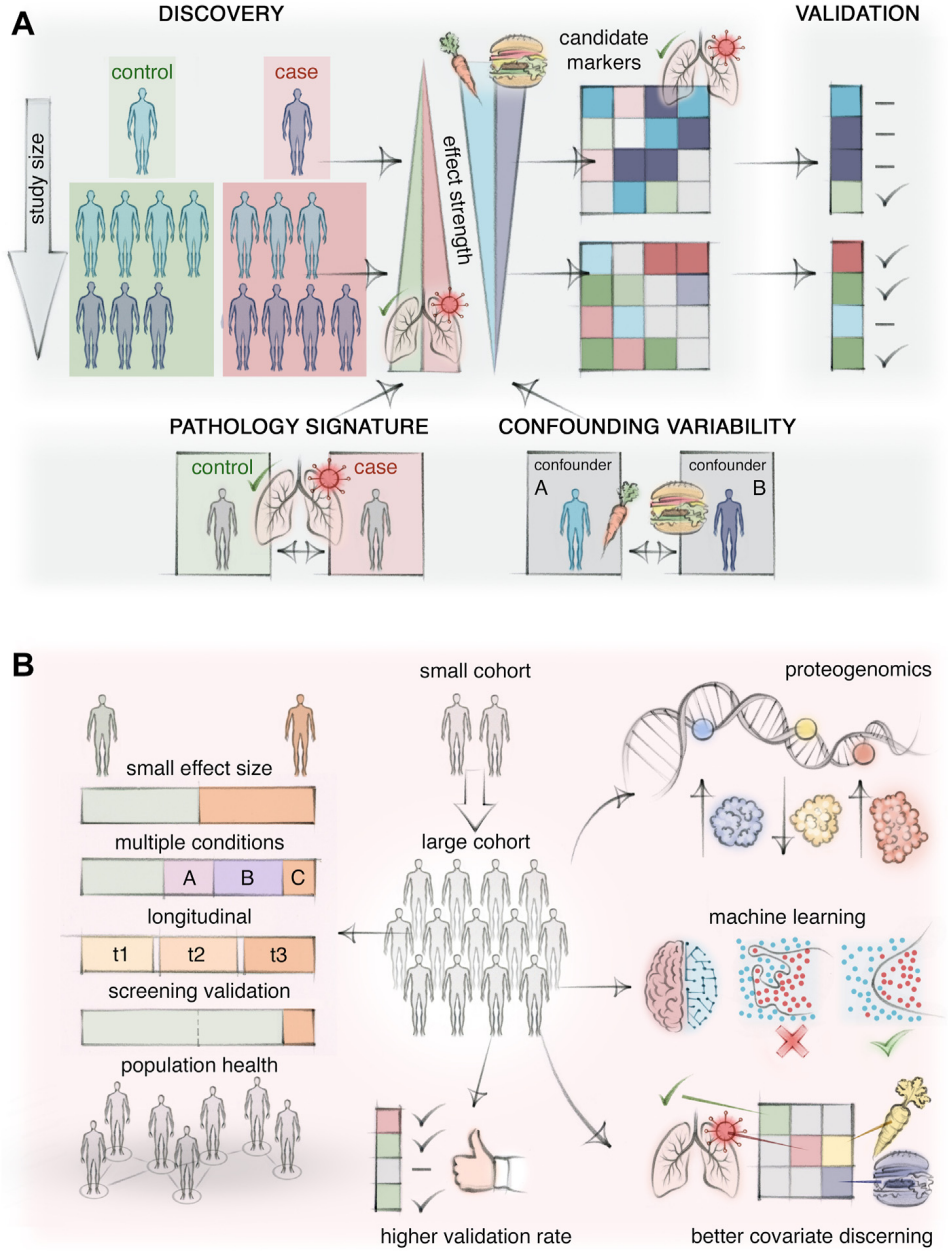
**Large cohort synergy with the rectangular strategy and additional benefits.***A*, Rectangular strategy harnesses large cohorts to better discover true biomarkers. Detecting biomarkers that distinguish pathological cases from controls faces challenges due to variability or biases introduced through confounders. Apparent proteome differences between cases and controls in small cohorts may be dominated by random confounding variability. This randomness dilutes with high numbers in large cohorts, leading to a dominance of true effects of interest and biomarker candidates with better prospects of validation in further independent cohorts. *B*, Large cohorts enable studying challenging scenarios. These include small effect sizes, comparison of multiple conditions for differential diagnosis or outcome analysis, longitudinal changes, validation of very high test accuracies for screening applications, or understanding phenotypes at the population level. Overall, large cohorts yield better biomarker candidates with higher validation probability and better resolve the effects of different covariates. Machine learning and in particular deep learning require high sample numbers to prevent overfitting of data. Finding associations between protein abundance levels and genetic variants requires many samples to reach statistical significance.

Despite mitigating measures, residual biases persist and pose a particular risk to small studies. The rectangular strategy instead harnesses large cohorts to provide the best chance to discern true biomarkers from non-generalizable candidates caused by confounding factors (Fig. 3*A*). Optimal selection of biomarker candidates for further validation is especially important, given the exponentially increasing costs when turning these candidates into actual clinical tests. Previously, sample throughput often limited study size, however, as noted, these constraints are rapidly disappearing. Our own studies were initially around dozens of samples but have moved on to hundreds of or even thousands of samples due to technological improvements (16, 17, 18, 19, 39, 110, 111). Although this still involves substantial efforts, we predict that this will become more commonplace soon. Thus, the size of a biomarker study will in the future be determined by what is epidemiologically desirable and practically feasible in terms of patient recruitment and biobank infrastructure. Given the large sample collection efforts, we believe that is crucial to maximize the information retrieved in terms of proteins measured, which is best achieved by the rectangular strategy with ever-increasing proteomic depth. Large study sizes and representative populations are also pivotal to enable certain forms of data analysis, most notably artificial intelligence. While classical machine learning has long been commonplace in biomarker studies in general and proteomics in particular, we believe that there is now great potential to apply the latest advancements in deep learning to biomarker discovery (112). Large and uniform datasets are an optimal starting point for such endeavors (Fig. 3*B*). Similarly, identification of genetic effects on protein levels in body fluids termed protein quantitative trait loci (pQTL) typically requires large study collectives (113). In MS-based proteomics, larger study sizes have drastically raised the number of identified pQTLs and there is a higher pQTL identification rate compared to similar affinity binder proteomics studies (94).

### Study Collectives and Analyses That Model Reality

Study collectives need to be matched to the studied disease and intended application. For instance, some diseases occur as a spectrum or continuum of phenotypes or manifest progressively, and controls may have different degrees of being at risk of or even affected by a disease. This may require longitudinal cohorts or comparing multiple conditions rather than relying on a binary classification into cases and controls (Fig. 3*B*). Additionally, complex scenarios may only be resolvable by a population-based understanding that is enabled by large study collectives. On the analysis side, researchers should not try to slice phenotypic continuums into ever smaller categories with specific biomarker signatures and cutoffs. Rather, biomarker panels should report a position on a continuum. Again, validation of the clinical utility of such findings hinges on the quality of clinical data. Apart from these issues, intended applications of biomarkers influence the study's collective composition. For instance, in screening applications, the specificity of a biomarker needs to reflect the underlying frequency of a condition in the population. To wit, if the general population is screened for a relatively infrequent condition, the specificity needs to be almost perfect while maintaining an overall high sensitivity to prevent the large majority of positive cases are actually false positives—a well-known issue for any population screen (11, 105). As a consequence, a high classification accuracy of a biomarker obtained in a cohort balanced for cases and controls alone does not demonstrate screening utility (99).

## Use Cases for Body Fluid Proteomics

### Measurement of Clinical Cohorts

**Fig. 4:**
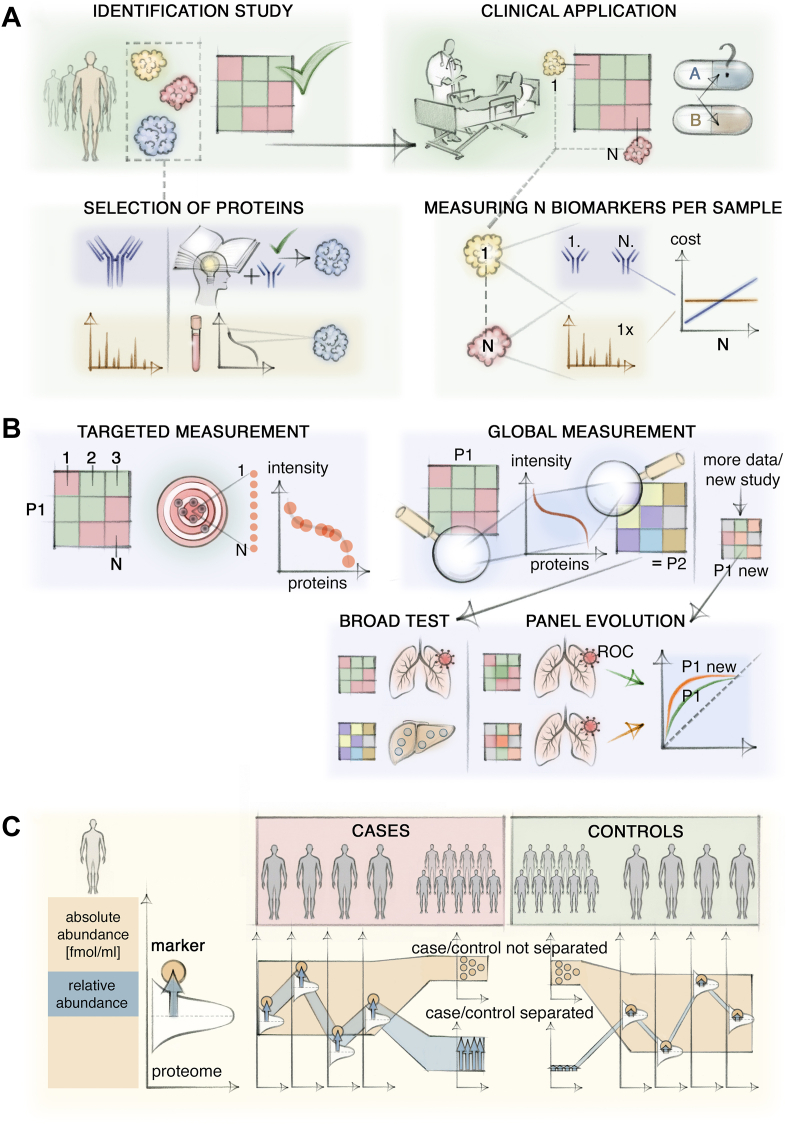
**Use cases and analysis concepts for MS-based proteomics.***A*, Strength of MS-based proteomics in biomarker discovery and routine application. In discovery, the unbiased nature of MS inherently leads to the identification and quantification of proteins in a “one fits all approach” that does not require prior insight or development of protein-specific reagents. Discovered biomarker panels comprising multiple proteins are quantified in routine applications to inform health care decisions. MS inherently scales well with the number of proteins with little extra costs. *B*, Targeted and global measurement approaches to quantify biomarkers. Targeting yields quantitative information only for the proteins that are actually targeted. Global approaches cover as large a part of the body fluid proteome as possible, yielding quantitative information on the biomarkers of interest. Extracting multiple biomarker panels for different questions makes this a broad multi-purpose test. Additionally, the proteomes measured in subsequent routine practice can be re-analyzed for refined versions of the initial biomarker panel. Second, improved software algorithms may enable deeper proteome depth from the same raw data, potentially enabling the identification of novel biomarkers that were previously unquantified. *C*, Quantification of biomarkers relative to the background or a reference proteome if used. This accounts for variability in the abundance of the entire proteome, including the biomarker, and can separate cases and controls even when variability in proteome abundance precludes such separation by absolute quantities.

In the clinic, the two roles of MS-based proteomics comprise the measurement of cohorts with the intent to find biomarkers and the much more common task of clinical routine measurement for diagnostic purposes (Fig. 4*A*). The unbiased nature of MS proteomics makes it well-suited for discovery without previous hypotheses. This approach also avoids the time-consuming establishment and validation of candidate approaches and does not require *a priori* knowledge of or insights into disease mechanisms. As technology improves, untargeted approaches such as DIA-based strategies with increasingly powerful mass spectrometers, will increasingly subsume the analytical capabilities of targeted MS assays (Fig. 4*B*). As mentioned earlier, MS-based proteomics competes with binder assays and should further develop throughput and proteomics depth to bring its inherent advantages to bear.

### Application in Clinical Routine

While there is great interest in using MS-based proteomics for discovery, its use in routine clinical diagnosis is much less established. This is in part due to perceived technological constraints such as throughput, robustness, and reproducibility of analysis. However, MS has made great strides in just the last few years: For instance, one-minute untargeted measurements of the plasma proteome enabled high-throughput and robust classification of the severity of COVID-19 (42). This throughput is already comparable to routine MS-based small molecule assays in clinical laboratories that typically involve measurement in a few seconds to minutes (114). Regarding reproducibility, median coefficients of variation below 20% have been shown for interday, untargeted proteome measurement in multiple academic laboratories across the globe (115). The analytical process of MS is compatible with automation and ISO norm quality management (15). Other hurdles for MS proteomics in routine settings are the high up-front costs for instrumentation and their complexity, currently requiring specialized staff for operation. The latter need not be a barrier forever as shown by the clinical laboratory use of MS for small molecule measurement which likewise entails higher complexity than immunoassays for setup, operation, troubleshooting, and analysis (116, 117, 118). The marginal costs of MS analysis are actually quite low, even down to one US dollar per sample in certain clinical laboratory settings for small molecules (118). MS also shines by analyzing many analytes in a single run, a severe limitation of established ELISA assays. This is even true in targeted MS assays, which are readily capable of measuring the typical number of proteins found in a biomarker panel (*e.g.* 5–20) (Fig. 4, *A* and *B*). This multiplexing and the high specificity and quantitative accuracy are key conceptual advantages of MS compared to classical ELISA or affinity binder technologies (118). In our experience, MS-based proteomics can also be much more tolerant of substances that may interfere with binding events such as triglycerides in patients’ plasma.

### Reference Proteome Alleviates the Need for Absolute Quantification

Absolute quantification, that is, pg/ml values in plasma, or some proxy of this is widely required in clinical practice to ensure interpretability, traceability, and transferability of measurements. Such absolute quantification in MS can typically be achieved by the addition of a known spike-in standard at some point in the workflow. However, this has proven to be quite challenging to control and does not necessarily cover the variability of prior steps such as digestion or pipetting variability. More generally, the notion of absolute concentration is more problematic than it first appears (Fig. 4*C*). For instance, urinary biomarkers/analytes are routinely normalized to urinary creatinine to ensure interpretability by accounting for water reabsorption in the kidney (119, 120). This demonstrates both that absolute concentrations are not always meaningful and second that use of normalized and thus relative abundances is an already accepted clinical practice.

In the creatinine case, physiologic dynamics in the excretion rate of creatinine introduce variability into this single-protein normalization (119). Experience in body fluid proteomics suggests that relative quantification with respect to the total body fluid proteome (possibly excluding some abundant but varying components) may be a more robust and interpretable measure for normalization than single-analyte normalization and in some cases even compared to absolute levels. Additionally, it has great analytical advantages: Relating analytes to the endogenous proteome can account for the variability introduced by sampling, storage, transport, and processing steps. If more stringent comparability is required, the above-mentioned reference proteome can provide this and can itself be absolutely quantified.

## Outlook

MS-based proteomics for body fluid biomarker discovery and clinical routine has evolved with the wide acceptance of DIA workflows, emerging technological innovations such as the integration of additional dimensions of ion separation, and the introduction of a reference channel in multiplexed DIA. These technological advancements increase throughput, robustness, and reproducibility. This expands potential applications by increasing throughput, aiding identification, quality control, performance monitoring, and cross-study integration. Coverage of urine, CSF, and similar body fluids is already excellent. Here MS-based proteomics is clearly competitive with large-scale binder assays, not least because the marginal costs of many MS-based workflows are becoming low due to their high throughput.

There are also promising developments for increasing proteome depth in plasma although this is work in progress. As we have shown, many of the challenges in body fluid proteomics have now been overcome and a number of studies have achieved verification in independent cohorts. Moreover, there is a proof-of-concept that the rectangular strategy with current cohort sizes and proteome depth can already yield biomarker panels with clinical utility, even outperforming state-of-the-art clinical assays in a study on liver disease. These biomarkers remain concentrated in the high abundance range, suggesting that many more await discovery as technology progresses and advances on multiple fronts are pushing the boundaries of detectable body fluid proteomes into lower abundance ranges.

We believe that MS-based body fluid proteomics is at a turning point and—with apologies to Churchill—we are at the “End of the Beginning” and rapid progress awaits. Large cohort sizes that used to present insurmountable challenges are now turning into great assets. The rectangular approach is now clearly feasible and successful with such cohorts, merging the previously separate discovery and verification stages, as long as independent cohorts are employed. This has already led to more generalizable biomarker candidates, thereby improving success rates for subsequent validation and clinical implementation.

Routine clinical application has traditionally been associated with technologies such as ELISA, but MS can contribute there as well and has unique advantages. Suitable routine detectability by MS is more likely if proteins were implicated as biomarkers by this technology in the first place. Marker panels are preferable to single protein markers as they better reflect the multilevel state of health, lifestyle, age, environment, and disease. Still, there are additional advantages of full proteome quantification for routine applications, such as the flexibility to interrogate the proteome for other questions, better quality control, and robust and meaningful quantification of proteins in relation to the background proteome. For the quantification of full proteomes and to some extent also for panels of markers, MS has an advantage as it scales well with the number of analytes in a given sample. Additionally, we argue that classical absolute quantification may neither be required nor desired in some cases, however, the use of a reference channel will also facilitate absolute quantification.

While global, unbiased analysis is clearly the method of choice for discovery, routine applications could in principle employ either global or targeted approaches. This is because it is increasingly possible to profile a large part of the proteome even in short analyses. Perhaps counterintuitively, the global measurements are inherently simpler as they are “one size fits all” regardless of the particular marker of interest, whose signal is simply extracted from the entire data set. Emerging hybrid approaches target a set of proteins of interest and also probe the global proteome, combining the advantages of both (121). The evolution of approved biomarkers highlights both the need and the potential for improvement. Re-analysis of a growing body of global proteomes, integrating additional samples over time and insight from other studies could speed up such an evolution. However, this faces challenges regarding privacy protection and can create ethical issues such as unsolicited information about “incidental finding,” that is unsuspected diseases (122, 123).

Clearly, more information would be better if the above issues could be resolved and routine, global body fluid proteome analyses would anyway be appropriate in routine health checks or wellness settings. Furthermore, measurement of the global plasma proteome in conjunction with phenotype information on a population level would be the ultimate beneficial application of plasma proteome profiling. Aggregation of such data sets could drastically increase the statistical power of biomarker associations, potentially leading to their comprehensive mapping. Although this sounds very speculative at this point, we note that analogous considerations and even pilot projects are well advanced in genomics, with a fully sequenced genome of every citizen a frequently invoked scenario.

In a broader vision, the proteomics field will also need to increasingly pay attention to non-technological aspects including developing infrastructures compatible with privacy protection regulation and greater communication with the medical communities. Biomarker needs and suitable study collectives should be jointly selected and prioritized. In that respect, the clinical utility has to be clearly defined and needs to be actionable, for example, by guiding therapeutic intervention or lifestyle changes. Additionally, the envisioned biomarkers and the action that it guides need to have a favorable risk-benefit and cost-benefit ratio for patients and the overall health care system. In practice, this can present a challenge if the benefit of the action cannot be studied without the biomarker being known first. This is a common concern when defining new molecular subtypes of a disease or diagnosis of a disease without effective treatment. The financial aspect is often underacknowledged but acts as a key barrier if there is no incentive for companies or healthcare players to carry biomarker candidates forward. Strong portable evidence in the form of multicentric verification is required to proceed to large-scale validation. Moreover, application in clinical routine will require regulatory approval, a lengthy and costly process. Cooperation with clinical laboratories, companies, and regulatory bodies, as well as funding agencies and reimbursement organizations, will be key for this mission. In this regard, key scientific values such as openness, transparency, and reproducibility (as embodied in the FAIR principles (124)) need to be balanced with GDPR (125, 126), HIPAA (127), and similar governmental requirements. Taken together, MS proteomics is poised to take on the challenge of body fluid biomarker projects from discovery to clinical routine, this time on better terms.

## Conflict of interest

M. M is an indirect investor in Evosep Biosciences.
